# Human Albumin Fragments Nanoparticles as PTX Carrier for Improved Anti-cancer Efficacy

**DOI:** 10.3389/fphar.2018.00582

**Published:** 2018-06-12

**Authors:** Liang Ge, Xinru You, Jun Huang, Yuejian Chen, Li Chen, Ying Zhu, Yuan Zhang, Xiqiang Liu, Jun Wu, Qian Hai

**Affiliations:** ^1^State Key Laboratory of Natural Medicines, China Pharmaceutical University, Nanjing, China; ^2^School of Pharmacy, Xinjiang Medical University, Ürümqi, China; ^3^Key Laboratory of Sensing Technology and Biomedical Instruments of Guangdong Province, School of Biomedical Engineering, Sun Yat-sen University, Guangzhou, China; ^4^Nanjing iPharma Technology, Co., Ltd., Nanjing, China; ^5^Department of Orthopedics, Xinqiao Hospital, Third Military Medical University, Chongqing, China; ^6^Guangdong Provincial Key Laboratory of Stomatology, Guanghua School of Stomatology, Hospital of Stomatology, Sun Yat-sen University, Guangzhou, China

**Keywords:** human serum albumin fragments, nanoparticle, paclitaxel, drug delivery, anticancer

## Abstract

For enhanced anti-cancer performance, human serum albumin fragments (HSAFs) nanoparticles (NPs) were developed as paclitaxel (PTX) carrier in this paper. Human albumins were broken into fragments via degradation and crosslinked by genipin to form HSAF NPs for better biocompatibility, improved PTX drug loading and sustained drug release. Compared with crosslinked human serum albumin NPs, the HSAF-NPs showed relative smaller particle size, higher drug loading, and improved sustained release. Cellular and animal results both indicated that the PTX encapsulated HSAF-NPs have shown good anti-cancer performance. And the anticancer results confirmed that NPs with fast cellular internalization showed better tumor inhibition. These findings will not only provide a safe and robust drug delivery NP platform for cancer therapy, but also offer fundamental information for the optimal design of albumin based NPs.

## Introduction

With the fast growing of material chemistry and nanomedicine, biodegradable nanoscale drug delivery platforms, including nanoparticles, micelles (Wang et al., 2014a; Li W. et al., 2016; Qu et al., 2017) and liposomes, have been widely utilized for biomedical diagnosis (Park et al., 2009; Morral-Ruiz et al., 2013; Wang et al., 2014b; Hu et al., 2018) and therapy (Boussif et al., 1995; Ding et al., 2013; Xing et al., 2013; Bertrand et al., 2014; You et al., 2016; Ge et al., 2017; Li et al., 2017; Pan et al., 2018; Yang et al., 2018). Recently, a great number of functional delivery systems (Liu and Lu, 2006; Cho et al., 2011; Shrestha et al., 2012; Wu et al., 2012, 2014; Li et al., 2013; Wu and Chu, 2013; Yu et al., 2013; Hai et al., 2014; Li H. et al., 2016; Hao et al., 2017; Xu et al., 2017) have been studied. But even the nanoparticles (NPs) based on FDA approved materials, such as the poly-𝜀-caprolactone (PCL), poly(DL-lactic acid), poly(lactide-cocaprolactone), and poly(lactide-co-glycolide) (PLGA), are still toxic for high dosage treatment (Singh and Ramarao, 2013). Then, NP systems with improved biocompatibility are highly desired (Maiti, 2011; Wang et al., 2016, 2018).

Albumin, as a biodegradable, non-toxic and non-immunogenic protein, has been used to prepare NPs (Elzoghby et al., 2012). Albumin based nanocarriers (Shimanovich et al., 2011; Altintas et al., 2013; Bakare et al., 2014; Rosenberger et al., 2014; Watcharin et al., 2014) have been reported and the albumin-bound paclitaxel (Abraxane^®^) had been proved to be safe and efficient (Saif, 2013; Cecco et al., 2014). The crosslinked albumin NPs were able to increase their physical stabilities, but the drug encapsulation efficiencies and sustained release still need to be improved (Kratz, 2008; Li et al., 2010, 2014; Elzoghby et al., 2012; Kou et al., 2018). For this goal, we hypothesized that albumin fragments based NPs could have better drug loading/release performance, which would result improved anti-cancer performance. Non-toxicity, biodegradability and preferential uptake in tumor and inflamed tissues make human serum albumin fragments (HSAFs) an ideal drug delivery system. Due to these advantages, it’s motivated to develop a novel and safety nanoplatform based on HASF.

**FIGURE 1:**
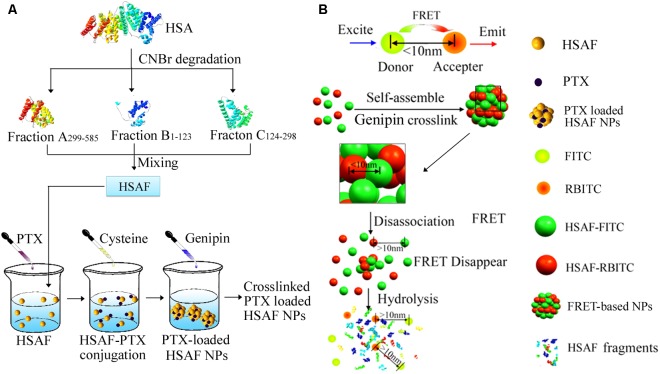
The illustration of nanoparticle preparation procedures and the related FRET phenomenon and the degradation process of NPs. **(A)** The fabrication of PTX-loaded HSAF NPs. **(B)** The degradation process of FRET-based NPs.

Therefore, in this report, as a model platform, a HSAF NP platform was developed as drug carriers with different crosslinking degrees and diameters using genipin as a very biocompatible crosslinker (**Figure 1A**). HSAFs were obtained via the degradation and the natural genipin crosslinker is expected to significantly reduce the toxicity while keep the similar crosslinking capability, comparing to the widely used glutaraldehyde. HSAF NPs were screened by a quantitative method based on FRET theory (**Figure 1B**) following previous report to obtain faster cellular uptake for further evaluations (Jiang et al., 2015).

## Materials and Methods

### HSAF NPs Preparation

Human serum albumin (HSA) was dissolved deionized water before 70% formic acid was added. CNBr was then added to degrade the HSA into fragment products (McMenamy et al., 1971). HSA degradation products were separated and purified by Superdex75 (Lapresle and Doyen, 1975; Yuan et al., 2014), and the three main peptide fragments are Fraction A_299-585_, Fraction _B1-123_, and Fraction C_124-298__(46-48)_ (w : w = 3.5:1:2.2), then the purified HSAF was used to prepare HSAF NPs. HSAF NPs were prepared and characterized according to the published protocols (Jiang et al., 2013). Then HSAF NPs with different crosslinking degrees and diameters were developed using a disulfide bond reducing method established in the previous work (Jiang et al., 2013). Briefly, a predeterminded amount of cysteine was added into the HSAF PBS solution under pH 8.0 at 37°C. The final concentration is 5 mg/mL. After dialysis, the remaining cysteine was removed and genipin was used to do second crosslinking (1 h). The genipin residue was cleaned by same method as above and the HSAF NPs were collected by lyophilization. The HSAF NP library with formulation parameters is summarized in **Table 1**.

**Table 1:** Physical and chemical properties of HSAF and HSA NPs.

	Crosslinking degree (%)	Diameter (nm)	Zeta potential (mV)	FRET Index (%)
C40S70_HSAF_	40.9 ± 1.5	65.9 ± 2.1	-20.86 ± 1.53	23.6 ± 0.9
C70S70_HSAF_	65.2 ± 2.0	68.0 ± 3.3	-24.09 ± 1.66	27.0 ± 2.8
C90S70_HSAF_	90.1 ± 0.9	74.7 ± 1.4	-27.12 ± 1.57	31.9 ± 0.4
C40S160_HSAF_	41.3 ± 1.0	157.3 ± 2.3	-23.58 ± 1.33	23.1 ± 0.3
C40S260_HSAF_	40.8 ± 1.1	255.9 ± 1.2	-20.13 ± 1.05	23.4 ± 1.3
C40S70_HSA_	38.8 ± 0.8	78.1 ± 0.3	-20.91 ± 1.51	23.7 ± 0.8
C70S70_HSA_	57.8 ± 1.9	80.1 ± 0.6	-23.09 ± 1.68	27.1 ± 1.7
C90S70_HSA_	85.8 ± 1.8	83.1 ± 1.1	-28.17 ± 1.59	30.6 ± 0.7
C40S160_HSA_	38.8 ± 1.1	182.7 ± 2.3	-24.11 ± 1.13	23.4 ± 0.6
C40S260_HSA_	37.6 ± 0.8	264.4 ± 3.1	-20.86 ± 1.53	23.5 ± 0.9

### Preparation and Characterization of Paclitaxel (PTX)-Loaded HSAF NPs

The PTX-loaded HSAF NPs with serious of crosslinking densities and diameters (PLC40S70, PLC70S70, PLC90S70, PLC40S160, and PLC40S260) were prepared following the same method preparing the above NPs. Briefly, the certain amount of HSAF was dissolved in PBS 8.0 at 37°C, and then PTX were dissolved in ethanol (PTX/HSAF50 mg/g) and genipin were added. Then system was incubated for 30 min to complete the crosslinking. The NP solution was purified and concentrated using Amicon Ultra Centrifugal Filters (MWCO 100,000). The size and zeta potential of PTX-loaded HSAF NPs were evaluated by a zeta potential and particle size analyser (ZetaPlus, Brookhaven, NY, United States). The morphology of nanoparticle was verified by transmission electron microscope (H-7650, HITACHI, Japan).

### PTX Drug Release Profiles of HSAF NPs

Dialysis was used to determine the release behavior of paclitaxel from nanoparticles. 3 mL of the nanoparticle suspension (containing 10 mg PTX) was placed in a dialysis bag (molecular weight cut-off: 13 kDa). The dialysis bags were placed in 80 mL of 1 M salicylic acid solution. Shaking was performed at a shaking speed of 100 rpm and a temperature of 37°C. 0.5 mL of dialysate was collected at 0.5, 1, 2, 4, 6, 8, 12, 24, 36, and 48 h, respectively, and an equal volume of fresh dialyzing media was added.

### Pharmacokinetic Studies

The PTX NPs (PLC40S70, PLC70S70, and PLC90S70) had similar diameters of about 70 nm but different crosslinking densities (41, 65, and 90%). And the NPs (PLC40S70, PLC40S160, and PLC40S260) prepared with albumin had similar crosslinking degrees around 42% but different diameters (65.9, 157.3, and 255.9 nm). The NPs (PLC40S70, PLC70S70, PLC90S70, PLC40S160, and PLC40S260) was i.v. administrated to Sprague-Dawley rats at a dose of 1 mg/kg as PTX. The blood sample was collected (100 μL) from rats into heparinized tubes at scheduled time (0, 0.083, 0.167, 0.333, 0.5, 1, 2, 4, 8, 12, and 24 h). Plasma was separated via centrifuging (4000 rpm, 10 min) and stored under -70°C until analysis. The drug concentrations were measured by LC–MS/MS (Wang et al., 2013) as previous study. For details, the analytes were eluted with at 5% mobile phase A methanol and 95% B water phase (containing 0.1% formic acid). The flow rate was 0.3 mL/min, and the temperature of column was 30°C. Mass analysis was operated in the positive ionization mode. Quantification was accomplished by monitoring the transition of m/z 876.0→307.8 for paclitaxel and m/z 830.3→549.0 for docetaxel (the internal standard). The spray voltage, the temperature of capillary, sheath gas pressure and auxiliary gas pressure were set at 4000 V, 350°C, 35 and 25 Arb, respectively. The pharmacokinetic parameters were estimated via a non-compartmental analysis (WinNon- lin computer program, Version 4.0; Pharsight Corporation). All the experiments were performed in accordance with the recommendations of “guidelines of the Experimental Laboratory Animal Committee of China Pharmaceutical University and the National Institutes of Health’s Guide for the Care and Use of Laboratory Animals.” The protocol was approved by the “Experimental Laboratory Animal Committee of China Pharmaceutical University.”

### *In Vivo* Imaging Study of HSAF Nanoparticles in Tumor-Bearing Mice

The NPs (C40S70, C70S70, and C90S70) had similar diameters around 70 nm but different crosslinking densities (42, 66, and 91%). And the NPs (C40S70, C40S160, and C40S260) prepared with various protein fragment concentrations had similar degrees of crosslinking around 40% but different size (65.9, 157.3, and 255.9 nm). DLC40S70, DLC70S70, DLC90S70, DLC40S160, and DLC40S260 expressed (DL means Dir was loaded) was made as the method as the NPs above, and the crosslinking densities and diameters differs not as the NPS without Dir. The certain amount of HSAF (Dir/protein 50 mg/g) was dissolved in PBS 8.0, and then the ethanol Dir was added at 37°C water bath. 5 mg/mL Cys was added. The system was cooled for 10 min. Genipin cross-linking was completed after dialysis. The configuration of the concentration of 50 μg/mL Dir which was dissolved in the polyoxyethylene castor oil and ethanol (50:50, V/V) solution was made as a control group. DLC40S70, DLC70S70, DLC90S70, DLC40S160, DLC40S260, and Dir solution formulation (0.5 mg/kg) was injected into the tumor-bearing mice via the tail vein. After intravenous injection, intraperitoneal injection of sodium pentobarbital solution (1%, 50 mg/kg) was given to anesthetize the mice. After the anesthetization, the whole body fluorescence images were acquired using small animal *in vivo* near-infrared imaging system at 0.5, 1, 2, 4, and 8 h.

### *In Vivo* Anticancer Evaluation in Breast Cancer Models

To evaluate *in vivo* anticancer activity of PTX-loaded HSAF and HSA NPs, PTX-loaded HSAF and HSA NPs were made by the method as C90S70 NPs whose particle size is the smallest and the crossing link degree is the highest. The 1 × 10^7^/ml MCF-7 cells were re-suspended in 9% saline, and 0.1 ml cells suspension was injected to nude mice on the right axillary subcutaneous. Tumor volumes were determined on alternate day by a vernier caliper, and the tumor volumes were calculated by an equation: V (cm^3^) = a × b^2^/2 (a: largest diameter; b: smallest diameter), meanwhile, mice weights were monitored three times per week. Fourteen days after tumor implantation, the volumes of tumor size were allowed to reach no less than 0.1 cm^3^, and mice groups (*n* = 8) were designed to have paclitaxel at dose of 5 mg/kg intravenously, (A) control group received 0.9% NaCl every 2 day (B) PTX Injection (PTX equivalent of 5 mg/kg) every 2 day; (C) HSA NPs (PTX equivalent of 5 mg/kg) every 2 day; (D) HSAF NPs (PTX equivalent of 5 mg/kg) every 2 day; After 28 days of initial treatment, mice were sacrificed and tumor tissues were collected. The tumor volume and weight were used for assessment of the therapeutic activity.

## Results and Discussion

### Synthesis and Characterization of Human Albumin Based NPs

**FIGURE 2:**
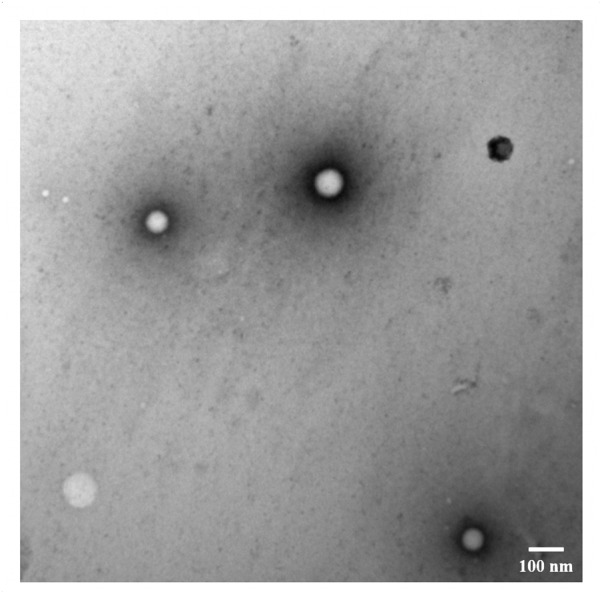
TEM image of C90S70 (length bar = 100 nm).

To figure out how physical and chemical properties of NPs may affect the cellular behavior of HSAF NPs, a HSAF NP platform was prepared using genipin as crosslinker with different crosslinking degrees and sizes, but similar surface charge (zeta potentials: -20 ∼-30 mV). The physical and chemical properties are summarized in **Table 1**. The NPs with different crosslinking degrees from 40.9 to 90.1% (C40S70, C70S70, and C90S70) were obtained by reacting with predetermined genipin. The NPs (C40S70, C40S160, and C40S260) prepared from different amount of albumin had similar crosslinking degrees around 41% but different sizes (65.9, 157.3, and 255.9 nm). These NPs are named as CxSy_z_: C means crosslinking; x means the crosslinking density; S means size; y means the NP size is around that number; z means is HSA or HSAF. A HSA or HSAF library could be obtained by varying the x and y. **Figure 2** showed the one example of TEM image of C70S70_HSAF._ The FRET indices of these NPs were in the range of 23 ∼ 32%, indicating that the NPs formulated in this study have significant FRET effects.

### Preparation and Characterization of PTX-Loaded HSAF NPs

The PTX-loaded HSAF and/or HSA NPs were prepared as the method as the NPs above. As shown in **Table 2**, compared to the HSA NPs, the smaller diameters and higher drug loading efficiencies were obtained by the HSAF NPs when using the same formulations.

**Table 2:** The diameters and drug loading efficiencies of the PTX-loaded HSAF and/or HSA NPs (*n* = 3).

	Diameter (nm)		PTX loading efficiencies (%)	
	HSAF NPs	HAS NPs	HSAF NPs	HSA NPs
C40S70	70.9 ± 3.3	87.2 ± 2.4	7.4	5.1
C70S70	74.0 ± 4.7	94.0 ± 3.6	7.2	5.3
C90S70	81.7 ± 3.2	95.7 ± 5.9	7.0	5.2
C40S160	172.3 ± 7.5	201.2 ± 15.8	8.3	6.5
C40S260	269.5 ± 14.6	295.1 ± 12.1	7.5	5.7

### PTX Drug Release Profiles of HSAF NPs

**FIGURE 3:**
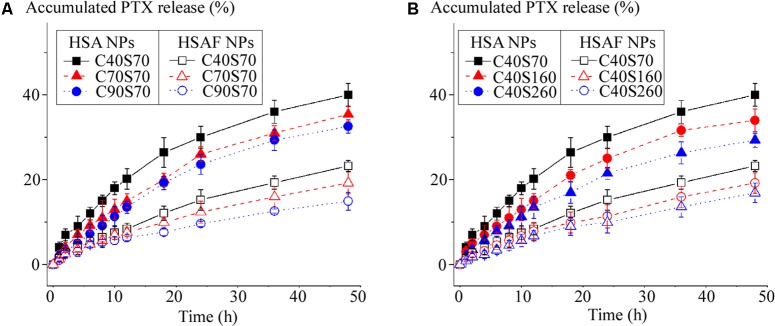
The cumulative release profiles of PTX from HSA NPs and HSAF NPs with various crosslinking degrees **(A)** and diameters **(B)** (*n* = 3).

*In vitro* PTX release profiles from HSA NPs and HSAF NPs were shown in **Figure 3**. Palitaxel release from NPs was detected by dialysis (Cho et al., 2004), the drug released was calculated at scheduled time (0.5, 1, 2, 4, 6, 8, 12, 24, 36, and 48 h). Compared with HSA NPs, PTX was released more slowly from HSAF NPs: within 48 h less than 50% of PTX was released from HSA NPs, but for HSAF NPs less than 25% PTX was released within the same time, indicating HSAF NPs could provide more possibility to delivery of PTX to specific organs and tissues than HAS NPs. For all HSA and HSAF NPs with comparable diameters, the increase of crosslinking degree decreased PTX release, perhaps because the compact structure of NPs, which was brought in by the chemical crosslink, hindered diffusion of PTX from NPs. F or NPs with comparable crosslinking degrees, the small NPs (C40S70 and C40S160) possessed faster PTX release behaviors, perhaps because small NPs possessed short drug diffusion distances.

### *In Vitro* Anti-cancer Evaluation of HSAF NPs

**FIGURE 4:**
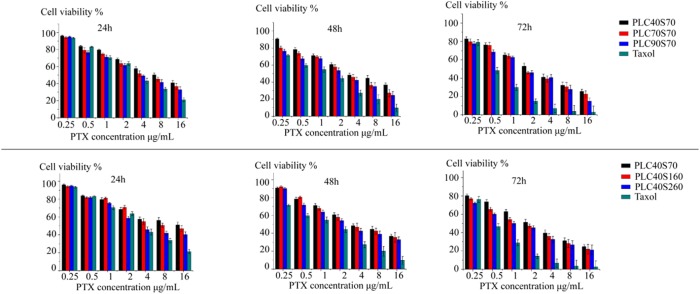
Cytoxicity of PTX-loaded HSAF NPs with different crosslinking degrees and diameters (PLC40S70, PLC70S70, PLC90S70, PLC40S160, and PLC40S260) against MCF-7 cells at 24, 48, and 72 h. The commercial product Taxol^®^ was used as the positive control (*n* = 6). PL, paclitaxel loading.

The cytotoxicity profiles of PTX-loaded HSAF NPs were compared in MCF-7 cells by MTT assay over a range of concentrations (0.25, 0.5, 1, 2, 4, 8, and 16 μg/mL). As shown in the **Figure 4**, the viability of the cells was dose-dependently decreased by the PTX-loaded HSAF NPs. Furthermore, the inhibitory effects of the NPs were increased with the NPs crosslinking density or diameter increasing at 24, 48, and 72 h, and this may result from the more efficient endocytosis brought in by the increased crosslinking degree and diameter. In comparison to the commercial product Taxol^®^, the weaker inhibitory effect of the PTX-loaded HSAF NPs was believed to associate with the sustained release and/or the relatively slower endocytosis of the NPs (Jia et al., 2014).

### Pharmacokinetic Studies of HSAF NPs

The major pharmacokinetic parameters of i.v. administration of HSAF PTX NPs have been summarized in **Table 3**. PTX NPs with lower particle size (C40S70, C70S70, C90S70) showed an obvious increasing in the AUC, MRT, t½ (*P* < 0.05), which correlated with an obvious decreasing in the Cl (*P* < 0.05). The results could be due to the lower particle size decrease the uptake of NPs by the mononuclear phagocyte system (Dobrovolskaia et al., 2008). The increasing of AUC, MRT, t½b and Cl could be achieved from C40S70, C70S70 and C90S70 as compared with C40S160 and C40S260 (*P* < 0.05), but the increment for C40S160 was not higher than C40S260. This result indicate that the NP size which is lower than 100 nm may be due to the inhibiting the fast uptake of NPs via the reticulo-endothelial system (RES) (Dobrovolskaia et al., 2008). The HAS PTX NPs which was made by the same method as C90S70 (C90S70HSA) was also studied, and the AUC, MRT, t½ of HAS PTX NPs is lower than that of HSAF PTX NPs (C90S70). There are significant difference between C90S70 and C90S70HSA (*P* < 0.05). The drug loading rate of C90S70 is higher than that of C90S70HSA, and the drug circulation time is longer than that of C90S70HSA too.

**Table 3:** Pharmacokinetic parameters of PTX after i.v. administration of NPs in mice (1 mg/kg).

Pharmacokinetic parameters	C40S260	C40S160	C40S70	C70S70	C90S70	C90S70HSA
AUC _0-t_ (μg h/L)	423.2 95.3	441.2 105.1	543.1 95.1*^#^	563.2 108.2*^#^	573.2 115.7*^#^	513.9 105.1
A UC _0-∞_ (μg h/L)	523.1 131.3	541.2 137.3	649.1 147.3*^#^	680.4 146.1*^#^	691.3 141.3*^#^	619.9 145.4
MRT _0-t_ (h)	2.3 0.4	2.6 0.7	4.40 0.9*^#^	4.54 1.2*^#^	4.58 1.3*^#^	4.13 1.1
Cl (L/h/kg)	1.9 0.4	2.0 0.5	1.4 0.5*^#^	1.3 0.4*^#^	1.2 0.6*^#^	1.5 0.5
V_d_ (L/kg)	28.4 9.7	29.4 12.1	31.4 12.4	32.48 11.9	38.4 12.1	30.4 11.3
C_max_ (μg/L)	1051.3 221.9	1067.3 233.9	1089.3 224.3	1099.31 224.6	1100.3 249.2	1087.1 219.8
t_1/2_ (h)	9.14 2.9	10.1 3.1	13.1 3.0*^#^	14.1 3.4*^#^	14.9 3.9*^#^	12.6 3.2

*Each value is the mean ± SD, n = 6. ^∗^P < 0.05, compared with C90S260; ^#^P < 0.05, compared with C70S160.*

### *In Vivo* Imaging Study of HSAF NPs in Tumor-Bearing Mice

**FIGURE 5:**
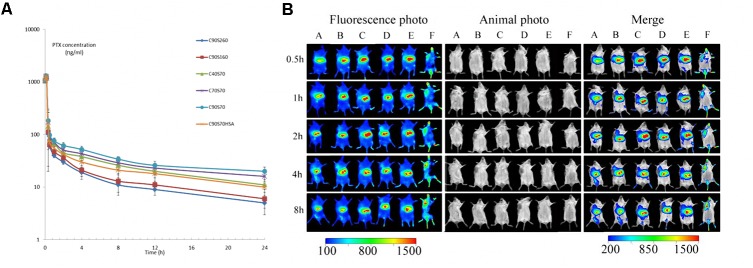
Paclitaxel plasma concentration profiles after intravenous administration of 1 mg/kg drug to Sprague-Dawley rats **(A)** and *in vivo* distribution of Dir labeled HSAF nanoparticles DLC90S70 _(A)_, DLC70S70 _(B)_, DLC 40S70_(C)_, DLC40S160 _(D)_, DLC40S260 _(E)_, and Dir solution _(F)_ in tumor-bearing mice **(B)**. Results are expressed with the mean ± SD (*n* = 6). DL, Dir loading.

As shown in **Figure 5**, most of the HSAF NPs containing the near-infrared fluorescent probe Dir clearly enriched in the liver after three kinds of HSAF NPs were delivered to tumor-bearing mice *in vivo*, indicating that the liver is still the barrier for NP system to achieve maximum efficient drug delivery to tumor, and how to avoid the NPs to be taken up by the liver is still priority problem for the drug delivery systems. The NPs with higher crosslinking densities or particle size distribute more to the liver. In addition to the liver, the right forelimb solid tumors in mice are the main distribution area of the HSAF NPs. From the graph, it could be observed that DLC40S70, DLC70S70, DLC90S70, DLC40S160, and DLC40S260 significantly concentrated in the tumor site at 4, 1, 0.5, 1, and 0.5 h, respectively. This difference suggests that NPs with higher size and the degree of crosslinking have the higher biodistribution to the tumor site. In this experiment, polyoxyethylene castor oil and ethanol (50:50, V/V) were used for preparing solutions. Dir was used as control, and we find Dir solutions distribute quickly to the mouse head, limbs, and solid tumors. The Dir solutions disappeared in the mice quickly and much faster than the nanoparticles.

### *In Vivo* Anticancer Evaluation of HSAF NPs for Breast Cancer Models

**FIGURE 6:**
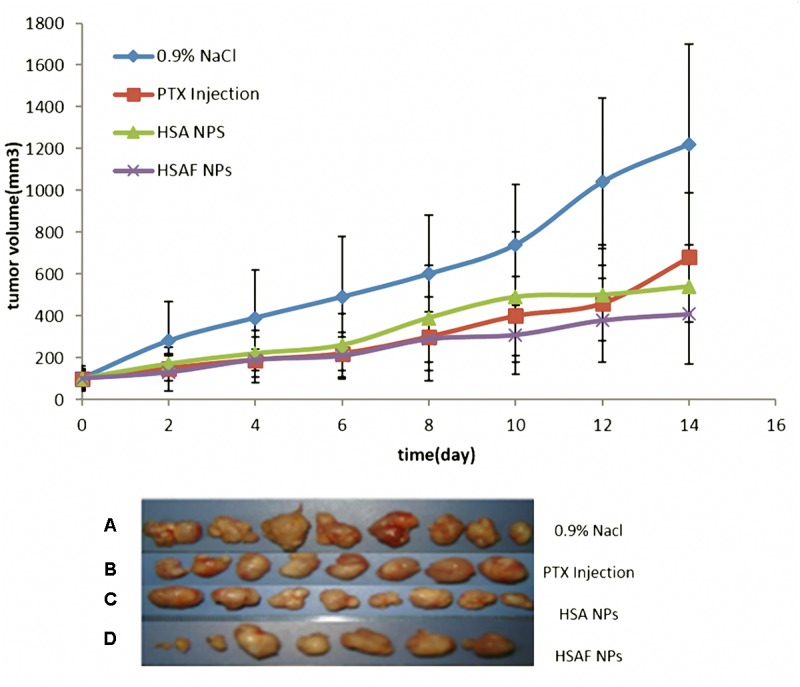
Picture of tumor and tumor size of four groups, **(A)** control group received 0.9% NaCl every other day **(B)** PTX Injection (PTX equivalent of 5 mg/kg) every other day; **(C)** HSA NPs (PTX equivalent of 5 mg/kg) every other day; **(D)** HSAF NPs (PTX equivalent of 5 mg/kg) every other day.

After 14 days of tumor inoculation, the average tumor volume was around 101 ± 23.19 mm^3^. Then administration was continued for a total of 28 days after tumor implantation. The results (**Figure 6**) showed that tumors were significantly (^∗^*P* < 0.01) inhibited after being treated with HSA NPs and HSAF NPs compared to PTX injection. Tumor inhibitory rate (tab.) in mice treated with HSA NPs and HSAF NPs were 63.3 and 71.4 respectively, which showed a significant (^∗^*P* < 0.01) compared to PTX injection and control groups. There was observed a non-significant (*P* > 0.05) change in tumor inhibitory rate for HSA NPs and HSAF NPs, and the tumor Inhibitory rate of HSAF NPs is higher than that of HSA NPs. There is no observable weight loss or other cytotoxicity in HSA NPs and HSAF NPs mice groups. Also, the tumor volume showed the same trend as the tumor weight, and the tumor volume of HSAF NPs group is the smallest. The dose of HSAF used in the NPS is lower than that of HSA, but the effect is better.

## Conclusion

In this report, a HSAF NP system with controllable crosslinking density and size were developed for better biosafety and anticancer efficacy. The HSAF NP library with a series of crosslinking degrees and particle sizes were developed, and the results showed that the similar particle size of HSAF NPs had different crosslinking densities, and the highest crosslinking density combined with the smallest particle size. This may lead to a higher drug loading rate and longer drug circulation time and further higher biodistribution in tumor site. Drug loading and release tests confirmed that the HSAF NPs have better drug loading and release performance than HSA NPs. *In vivo* anticancer evaluations confirmed that the NPs with fast cellular uptake showed better tumor accumulation and tumor inhibition. The results provide basic information not only for the biochemical effects and biosafety of albumin based NPs, but also for regulating the physicochemical properties which are important for the *in vivo* delivering of drugs.

## Author Contributions

LG, JW, and QH conceived and directed the study. YC and LC prepared NPs and obtained spectroscopic results. LG and XY co-wrote the paper. LG, XY, and JH contributed to the results analysis and discussion. YiZ and YuZ provided technical support and corrections of manuscript. XL, JW, and QH oversaw the project. All authors reviewed and approved the final paper.

## Conflict of Interest Statement

YC was employed by company Nanjing iPharma Technology, Co., Ltd. The other authors declare that the research was conducted in the absence of any commercial or financial relationships that could be construed as a potential conflict of interest.
